# Antagonism of Propofol Anesthesia by Alkyl-fluorobenzene Derivatives

**DOI:** 10.21203/rs.3.rs-3846123/v1

**Published:** 2024-01-11

**Authors:** Diana M. Plasencia, Liam H. Rodgers, Alexys R. Knighton, Roderic G. Eckenhoff, E. Railey White

**Affiliations:** 1 Department of Anesthesiology & Critical Care, University of Pennsylvania, Perelman School of Medicine, Philadelphia, United States of America

## Abstract

Despite their frequent use across many clinical settings, general anesthetics are medications with lethal side effects and no reversal agents. A fluorinated analogue of propofol has previously been shown to antagonize propofol anesthesia in tadpoles and zebrafish, but little further investigation of this class of molecules as anesthetic antagonists has been conducted. A 13-member library of alkyl-fluorobenzene derivatives was tested in an established behavioral model of anesthesia in zebrafish at 5 days post fertilization. These compounds were examined for their ability to antagonize propofol and two volatile anesthetics, as well as their binding to the anesthetic-binding model protein apoferritin. The two compounds demonstrating highest antagonistic potency were found to bind apoferritin in a manner similar to propofol. Selected compounds did not show antagonism of volatile anesthetics, indicating some selectivity of this antagonism. Similarities in structure and binding to apoferritin as well as a Schild analysis are suggestive of competitive antagonism, but like the anesthetics, the potential mechanism(s) of these antagonists will require further mechanistic investigation.

## Introduction:

Propofol is widely used for induction of anesthesia and has gained increasing popularity as a maintenance anesthetic for many procedures, from colonoscopies to craniotomies, as well as sedation in intensive care units (ICUs).^[1]^ The benefits of propofol include its relatively fast onset and offset of action as well as a comparatively low side-effect profile; however, propofol has non-anesthetic effects (including cardiovascular and respiratory depression) that can result in catastrophe if not anticipated and mitigated. One approach to mitigation would be drug antagonists. With the introduction of sugammadex, there are now antagonists for every class of commonly used perioperative drug except anesthetics.^[2]^ There are many clinical settings in which an anesthetic antagonist could be useful. In addition to saving time during emergence, an antagonist could be lifesaving when seconds count, such as the dreaded ‘can’t ventilate, can’t intubate’ scenario. Further, an antagonist may facilitate critical neurological evaluations in the ICU, and obviate or expedite the need for a CT scan to differentiate a stroke from residual anesthesia. These are just a few examples where the availability of an anesthetic antagonist could change clinical practice.

Prior efforts to identify possible anesthetic antagonists have suffered from an incomplete knowledge of anesthetic molecular pharmacology.^[3]^ Given that many anesthetics activate the GABAA receptor (GABAAR), rational drug design of GABAAR inhibitors have been pursued with some success^[4]^, but translation to in vivo anesthetic antagonism is lacking. Others have focused on non-specific CNS stimulants (caffeine, methylphenidate, etc.) to hasten the emergence from anesthetics,^[5–7]^ though undesirable side effects (dysrhythmias, hemodynamic extremes, delirium, psychosis, etc.) can occur.^[8,9]^ Furthermore, these nonspecific drugs are less effective for propofol, the most commonly used anesthetic for induction and sedation. An effective antagonist specific to propofol with few side effects would be clinically useful, and a logical approach may be to explore propofol derivatives.

The study of anesthetic structure activity relationships (SARs) dates back to Meyer and Overton in the 1880s and has continued as a means of anesthetic optimization, as well as to probe potential anesthetic mechanisms.^[10,11]^ Propofol derivatives have previously been classified as either “active” or “inactive” as anesthetics, where the inactive ones are disregarded.^[12,13]^ But a lack of anesthetic activity does not necessarily correspond to lack of any pharmacologic activity. For example, the molecule Fropofol (fluorinated propofol),^[14]^ (renamed propofluor for improved phonological clarity), is one such molecule. Although propofluor is inactive as an anesthetic, it was found to antagonize propofol in tadpoles^[14]^ and zebrafish.^[15]^ This study examines alkyl variations of propofluor for anesthetic and antagonistic properties using a previously validated zebrafish behavioral model of anesthesia.^[16,17]^

## Results:

### Selection of library compounds.

**Figure 1.**
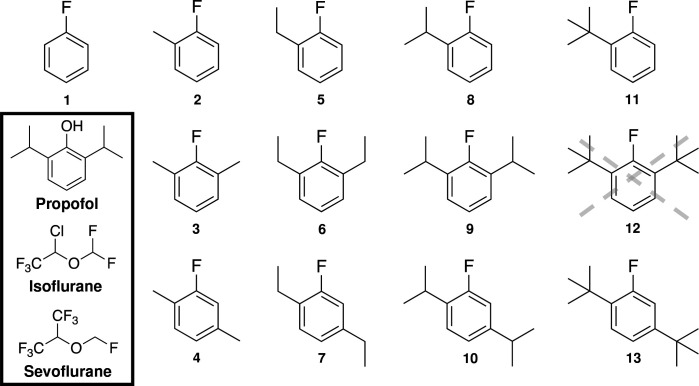
Chemical Structures of the Propofluor Arm Library (PFAL) Compounds and Commonly Used Anesthetics. This library of compounds is composed of alkyl derivatives of the molecule propofluor (compound **9**, previously called fropofol). The intended compound 12 was unable to be synthesized and thus not characterized. Chemical names and abbreviations are as follows: **1**: fluorobenzene (FB), **2**: 1-methyl-2-fluorobenzene (1-Me-2-FB), **3**: 1,3-dimethyl-2-fluorobenzene (1,3-diMe-2-FB), **4**: 1,4-dimethyl-2-fluorobenzene (1,4-diMe-2-FB), **5**: 1-ethyl-2-fluorobenzene (1-Et-2-FB), **6**: 1,3-diethyl-2-fluorobenzene (1,3-diEt-2-FB), **7**: 1,4-diethyl-2-fluorobenzene (1,4-diEt-2-FB), **8**: 1-isopropyl-2-fluorobenzene (1-iPr-2-FB), **9**: 1,3-diisopropyl-2-fluorobenzene (1,3-diiPr-FB) **10**: 1,4-diisopropyl-2-fluorobenzene (1,4-diiPr-FB), **11**: 1-tertbutyl-2-fluorobenzne (1-ditBu-FB), **12**: 1,3-ditertbutyl-2-fluorobenzene (1,3-ditBu-FB), **13**: 1,4-ditertbutyl-2-fluorobenzene (1,4-ditBu-FB). The chemical structures of 3 commonly used anesthetics (Propofol, Sevoflurane and Isoflurane) are shown in the lower left corner.

All compounds for the propofluor arm library (PFAL) were designed around a fluorobenzene core, with variable alkyl ‘arms’. One could generate a massive library solely with modifications to alkyl carbon number, position, and shape, but the final 13-member PFAL (Fig. 1) consisted of a subset of non-chiral alkyl arms (methyl, ethyl, isopropyl and tertbutyl) in three different positions (1, 1,3 and 1,4) on the fluorobenzene ring to create a library with systematic alterations in size and configuration of the alkyl carbon side chains. The proposed compound **12** (1,3-diturtbutyl-2-fluorobenzene) was unable to be synthesized and thus not included in the library. Physiochemical parameters for these compounds can be found in Table S1.

### Adverse effects of the PFAL compounds.

**Figure 2.**
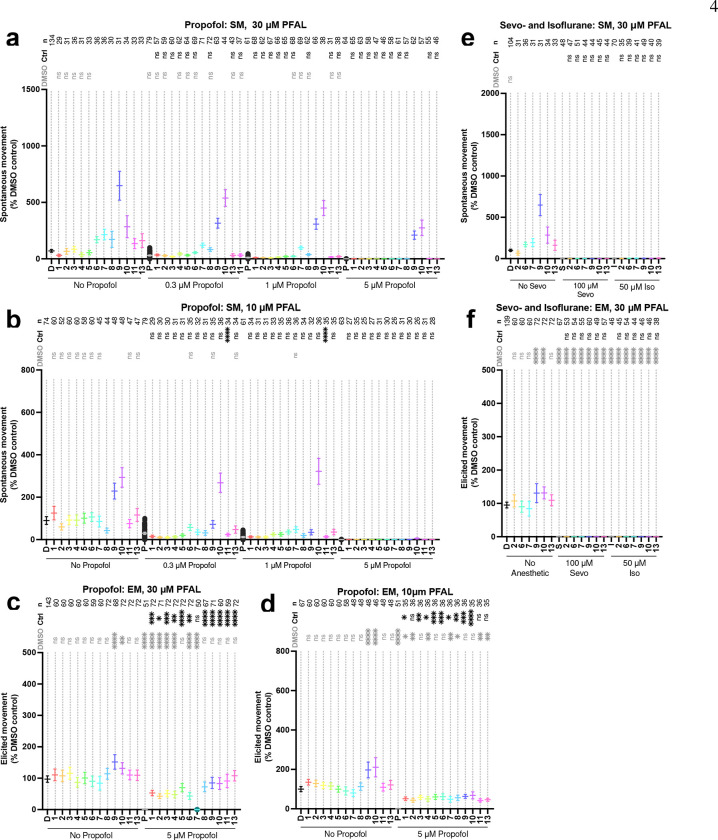
Screening of PFAL Compounds for Antagonism of Anesthesia. PFAL compounds were screened in 5dpf zebrafish for their ability to antagonize the anesthetic effects of propofol (panels a, b, c, and d) as well as sevoflurane and isoflurane (panels e, and f). Each dot represents the data collected from one fish. Spontaneous movement (SM) is the distance of spontaneous swimming in a 4-minute period, and elicited movement (EM) is distance moved in 1 second after exposure to a tap stimulus. Both measures were scaled to same-day DMSO controls. Concentrations of drugs used for screening were based on previously determined EC_50_ values: Propofol SM = 0.01 μM, EM = 2.8 μM; Sevoflurane SM = 76 μM, EM = 240 μM; Isoflurane SM = 42 μM, EM = 180 μM.^[16]^ Mean and 95% CI are shown for each data set. Statistical comparison with both DMSO (D) only (black asterisks) and anesthetic control (Ctrl, gray asterisks) groups are shown. Note that each DMSO comparison is to the DMSO only control (sample on the furthest left of each graph, and the anesthetic control comparisons are made within each anesthetic grouping as indicated by the bars along the x-axis of each graph. ns: P > 0.05, *: P ≤ 0.05, **: P ≤ 0.01,

The first step in the testing of this library was an assessment of the toxicity. The small size of 5 pdf (days post fertilization) larval zebrafish (<4 mm total body length) facilitates bulk diffusion for gas transport, thus toxicity derived from respiratory depression is inaccessible and therefore a limitation of this model compared to adult fish, and mammals that rely on breathing and bulk flow respiration.^[18,19]^ Nevertheless, an LD_50_ (median lethal dose) in this model remains a useful means to compare toxicity via, but also prevents mistaking morbidity or mortality for anesthesia. In this assay, PFAL compounds were found to be considerably less toxic than propofol itself (Fig. S1), including some with LD_50_ values that were unable to be determined due to limited solubility. For these compounds (**1-5**) an LD_50_ was estimated with available experimental data and an upper constraint of 100% mortality. All concentrations of PFAL compounds used in later testing were far below these LD_50_ values. Co-administration 30 μM PFAL compounds with propofol yielded a minimal increase (<6 μM changes) in toxicity that remains far above propofol’s clinically useful concentrations (Fig. S1). Patterns of spontaneous movement (SM) can also be used to screen for adverse behavioral phenotypes, such as seizure (pentylenetetrazole was used as a positive control for seizure, see Supplemental Video).^[20]^ Compounds **9** and **10** demonstrated an increase in movement in a dose-dependent fashion (Fig. 2); however, no PFAL compound elicited movement patterns indicative of seizure or other atypical phenotypes (Fig. S2 and Supplemental Video).

### Sedative activity of the PFAL compounds.

Each compound was tested for sedative activity when administered alone prior to combination testing with propofol. PFAL compounds alone at 10 and 30 μM revealed no statistically significant decrease in SM or elicited movement (EM) that would indicate sedation or anesthesia (Fig. 2 a–d). Although no decrease in movement was observed, this test alone does not rule out sub-clinical sedation that might be observed in an additivity study with propofol.

### Antagonism of propofol anesthesia.

Based on previously reported EC_50_ values in 5 dpf zebrafish,^[16]^ propofol (0.3, 1, and 5 μM) was co-administered with PFAL compounds (up to 30 μM) as an initial test of anesthetic additivity or antagonism. Compounds **9** and **10** administered at 30 μM showed consistent antagonism of propofol up to 5 μM. At 10 μM, **10** was able to antagonize a very high concentration of propofol (1 μM) which is 10-times higher than propofol’s SM EC_50_ of 0.1 μM. This initial screen suggests compound **10** may exhibit increased potency as a propofol antagonist compared to the previously characterized compound **9** (propofluor).

### Antagonism of sevoflurane and isoflurane anesthesia.

To assess the selectivity of this anesthetic antagonism, a smaller group of PFAL compounds was tested for antagonism of the volatile anesthetics sevoflurane and isoflurane. As with propofol, anesthetic concentrations were chosen for each drug based on previously reported EC_50_ values,^[16]^ and PFAL doses of 30 μM. No evidence of antagonism of sevoflurane was found and only compound **10** showed modest antagonism of isoflurane at a concentration of 25 μM (Fig. 2e–f). These findings suggest some specificity for antagonism of propofol anesthesia, consistent with previous findings that suggest an inverse to this relationship (some stimulants having greater potency in antagonizing volatile anesthetics compared to propofol),^[7]^ and is further evidence for underlying differences in mechanisms of action for these anesthetic classes.

### Antagonism of anesthesia after induction.

**Figure 3.**
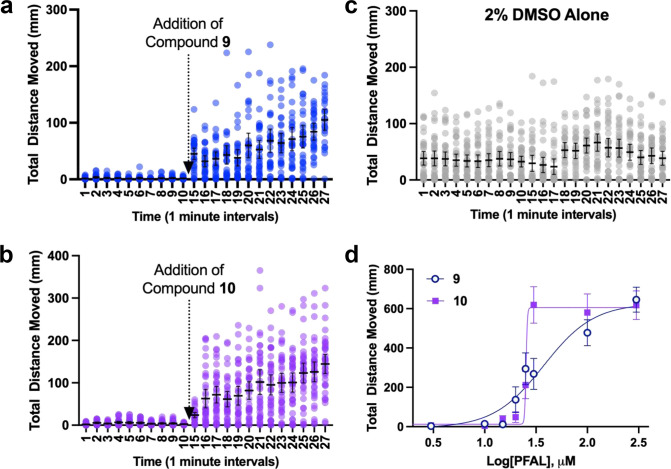
Further Characterization of Propofol Antagonism with Compounds 9 and 10. a and b) 5dpf zebrafish (n = 36) were first anesthetized with 3 μM propofol and then 30 μM of either compound **9** or **10** was added. Between minutes 5 and 10 there is a small gap in the data during which the propofol solution was removed and a solution containing propofol and PFAL compound was added. Spontaneous movement of the fish resumed very shortly after compound addition. c) 2% DMSO vehicle control for panels A and B. n = 36 for panels A-C (each dot represents 1 fish). d) IC_50_ of compounds **9** and **10** measured in 5 dpf zebrafish exposed to 3 μM Propofol was found to be 37.5 μM and 25.3 μM respectively. The mean and 95% CI is shown for each data point in all panels.

In the above studies, the anesthetic and PFAL compounds were co-administered to the fish simultaneously. In order to show that these compounds can antagonize ‘anesthesia’ once established, a sequential addition was conducted whereby the fish were exposed to propofol (3 μM) for 10 minutes, the solution was removed and replaced with solution containing propofol (3 μM) and either compound **9** or **10** (30 μM). Although precise kinetic data was not generated, it is clear that the exposure to either **9** or **10** produced rapid reversal of propofol immobility despite the continued presence of an immobilizing concentration of propofol (Fig. 3a–c).

### IC_50_ of compounds 9 and 10.

The initial screening experiments revealed **9** and **10** as the most potent anesthetic antagonists in the PFAL library. At a propofol concentration of 3 μM, IC_50_ (median inhibitory concentration) values were determined to quantitatively compare their potency (Fig. 3d). The IC_50_ values were closer than the initial screening experiments suggested (IC_50_ of **9** = 37.5 μM, **10** = 25.3 μM); however, the finding is less surprising when one considers the striking difference in Hill slopes (**9** = 1.8, **10** = 65), may also be an initial clue to that these compounds may have mechanistic differences.

### Inhibition of 1-AMA binding to a model protein.

**Figure 4.**
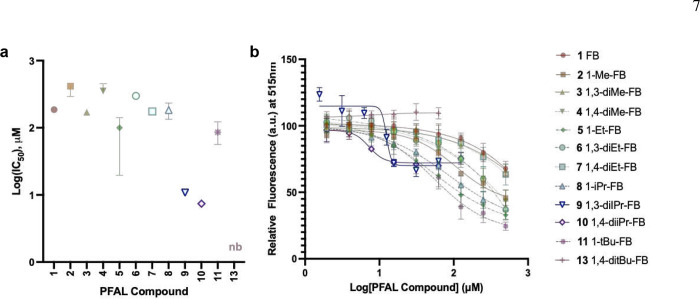
Binding of PFAL Compounds to Apoferritin. The relative binding affinity of PFAL compounds to HSAF (horse spleen apoferritin) was measured by displacement of the fluorophore 1-AMA (1-aminoanthracene) that demonstrates increased fluorescence when bound to HSAF. Error bars represent 95% CI of the calculated IC_50_, but upper limits were unable to be estimated for compounds **1**, **3**, **4**, **6**, or **7**. nb = non-binding a) Calculated IC_50_ of PFAL compounds. b) Binding curves from which IC_50_ values were calculated. Mean and standard deviation for each data point are shown.

**Figure 5.**
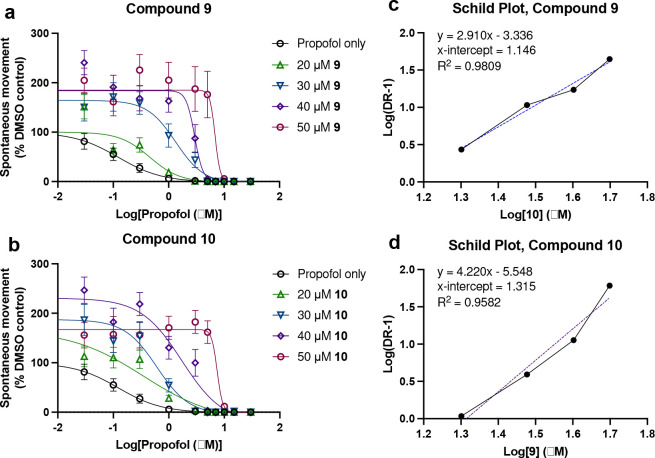
Schild Analysis of Propofol Antagonism with Compounds 9 and 10. a and b) EC_50_ curves of propofol (SM) with co-administration of compounds 9 and 10 at 0, 20, 30, 40, and 50 μM. Error bars indicate 95% CI. c and d) Schild plots derived from the EC_50_ data in sub-panels a and b.

The ability of anesthetics, to inhibit 1-aminoanthracene (1-AMA) binding to the ‘anesthetic site’ of model protein HSAF (horse spleen apoferritin) has been shown to correlate to anesthetic activity.^[21–23]^ However, it is known that **9** also binds to this site,^[14]^ suggesting the assay may also reveal optimal physicochemistry of other antagonists. Each compound in the library inhibited the binding of 1-AMA to HSAF (indicated by decreased fluorescence from unbound 1-AMA), except for compound **13** where testing was limited by solubility (Fig. 4). The calculated IC_50_ values would suggest that compounds **9** and **10** are the most potent inhibitors of 1-AMA binding (Fig. 5A). However, the shape of these IC_50_ curves for **9** and **10** are markedly different from the other PFAL library members in that they demonstrate incomplete inhibition of 1-AMA binding which is similar to the binding seen by propofol in this system (Fig. S3). This similarity to propofol is consistent with the hypothesis of competitive antagonism of propofol. The appearance of incomplete competition at soluble concentrations is not explained by 1-AMA/PFAL interactions (Fig. S4) and is not readily explained by our current understanding of anesthetic binding in this system.

### Schild Analysis.

To begin an initial investigation of potential mechanisms of action, a Schild analysis was conducted for both compounds **9** and **10**. EC_50_ curves for propofol with the addition of 0, 20, 30, 40, and 50 μM **9** and **10** were obtained (Fig. 5a–b), and then used to form the basis of Schild Plots (Fig. 5c–d). Compound **9** exhibits linear correlation suggestive of competitive binding, while the Schild plot for **10** has a slight curvature that may indicate some level of cooperative binding.^[24]^ However, such an analysis would require all assumptions of a Schild analysis be met,^[25]^ which may not be the case in this system given the unknown receptor(s) mediating this response. The slope of the linear fit is also >1 for both compounds. This is often attributed to non-specific binding, often adsorption to glassware, but given the known non-specific binding of propofol in complex biological systems it is not surprising that these molecules would behave similarly. This analysis must be viewed with caution as even if compounds **9** and **10** act at a single site, propofol most likely does not which could explain the observation that these antagonists appear to be surmountable antagonists and thus do not exhibit the classic “parallel shift” seen for a classic competitive antagonist.

## Discussion:

In this study, we have found that many fluorobenzene derivatives of propofol possess rapid antagonistic activity to propofol anesthesia in a larval zebrafish model. Differences in antagonist potency based on alkyl “side arms” exist and appear to correlate to some degree on hydrophobicity, but size/shape and steric hindrance must also be considered. Strikingly little antagonism of two volatile anesthetics was apparent, suggesting antagonism is not due solely to non-specific CNS stimulation.

Like the mechanism(s) of anesthesia generally, the mechanism(s) of antagonism by these alkyl-fluorobenzenes is not clear. It would be logical to propose that molecules that are structurally similar to propofol, would act by competitive antagonism, but competitive binding to what target(s) remains unknown. Anesthetics are known to be ‘promiscuous’, interacting with many targets, and thus the possible targets or their combination mediating this effect could be large in number. This may also be why these compounds appear to be ‘surmountable’ antagonists. Despite increasing concentrations of **9**/**10**, there is consistently zero movement observed by approximately 10 μM propofol and a corresponding increase in hill slope with increased PFAL concentration (see Fig. 5a–b). The GABAAR is most prominently implicated in anesthetic mechanisms,^[26]^ but compound **9** has previously been shown to produce no significant modulation of a propofol-sensitive GABAAR.^[14]^ While the linearity of the Schild plots might suggest competitive antagonism, consistent with the hypothesis based on structural and physicocochemical similarity, we can concluded based on previous findings that this phenomenon is not GABAAR-mediated.

Because of the absence of validated in-vivo molecular targets, surrogate targets were employed to study anesthetic protein binding. Principal among them have been firefly luciferase^[27]^ and apoferritin,^[28,29]^ both of which have crystal structures with bound anesthetic. However, they are less than ideal models because each bind compounds that do not produce anesthesia, as shown here for apoferritin. However, it is curious that antagonistic compounds **9** and **10** have similar binding curves as propofol (Appendix Fig. 8) where a calculated EC_50_ values show higher affinity in part due to incomplete competitive binding. In a screen for anesthetics using HSAF as a model for anesthetic binding, one might erroneously conclude that **9** and **10** would be anesthetics.^[22,23]^ However, the similarities in binding of **9**, **10**, and propofol suggest they interact with HSAF in a similar manner that is different from the other PFAL compounds that were able to compete with 1-AMA (albeit with a lower binding affinity), but are neither anesthetics nor effective antagonists. These results suggest that binding to HSAF without further interpretation is not an ideal anesthetic model but is does provide a physicochemical model that can distinguish propofol and its antagonists from other less active molecules.

Anesthetic agents across many species are known to have steep population dose-response curves,^[30–32]^ so it is not unreasonable to expect an anesthetic antagonist to have a similar feature. The basis for these steep dose response relationships is debated, but one explanation relies on complexity, or the number of contributing molecular targets.^[26,30,32,33]^ Certainly, the most potent antagonist **10** appears to possess this feature, with a very steep dose response relationship. The more gradual change in antagonistic effect of **9** is typical of more specific pharmacologic agents. Combined with **9**’s close structural similarity to propofol, this may indicate more close competitive antagonism at one or more sites. The steep slope observed with the EC_50_ curve of **10** (Fig 3d) likely indicative of differences in binding between **9** and **10** despite their seemingly subtle differences in carbon-backbone. This slope is so steep, the flip between anesthetized states happens like a ‘switch’, which is not characteristic of competitive antagonism, and thus also supports potential mechanistic differences brought about by the subtle differences between these compounds. The mechanistic differences between these antagonists might provide clues into both anesthetic and antagonist mechanisms and reveal potentially new druggable targets.

The alkyl-fluorobenzene molecules tested in this study are just a small sampling of the vast number of possible configurations of an alkylphenol or alkyl-fluorobenzene. Recent work on ciprofol highlights how even a seemingly small number of carbon arrangements and chirality centers can become both increasingly complicated, but have a significant effect on potency.^[34]^ However, a potentially more interesting avenue of further study might be to instead alter electronic configurations to further elucidate the key features differentiating anesthetics and antagonists or even other “inactive” molecules with yet uncharacterized biologic effects. By augmenting or diminishing certain features of propofol, it may be possible to fine tune the functionality of these molecules that interact with likely many untold targets.

A principal limitation one might posit in this study is the non-mammalian model used to determine activity. However, the larval zebrafish model is a vertebrate that is phylogenetically closer to mammals than other anesthetic models such as the fly and worm. They also exhibit complex behaviors, and have even demonstrated learning, and their anesthetic sensitivity has been shown to correlate to mammals.^[16,35–37]^ Nonetheless, the zebrafish is a genetically heterogeneous model, and thus can have wider variation in their responses to a given condition compared to rodent models. Additionally, the larvae are still developing, and like mammals, anesthetic potency changes as a function of age. Thus, the consistency of these findings in other developmental stages, and the translation of these results to rodent models are important areas of future investigation.

Efforts to elucidate the yet unidentified targets for both propofol and these PFAL compounds, is an important area of future investigation. Nevertheless, even without a clear mechanism, the ability to reverse propofol-induced anesthesia could prove to be an important control in studies from the molecular to network levels, and like the ‘non-immobilizers’,^[38]^ adds to the potential toolbox for understanding anesthetic actions.

In summary, we describe an innovative approach to terminating anesthetic activity, and provide a screen of alkyl-fluorobenzenes as one potential propofol antagonist chemotype. In the larval zebrafish model, we found that these compounds are antagonistic to propofol-induced immobility. Further, even the most potent propofol antagonists do not antagonize volatile anesthetic-induced immobility. This finding along with the initial pharmacologic investigations presented here, support a hypothesis of competitive antagonism. Like anesthesia itself, mechanisms of antagonism are yet to be revealed, but are unlikely to be as simple as competitive binding at a single site. This is likely only a small subset of molecules that antagonize propofol anesthesia, and additional improvements in potency will further aid in mechanistic investigations that underpin these curious findings.

## Methods:

### Library compounds.

All compounds were purchased from commercial sources including ‘make on demand’ custom synthesis via ChemSpace: **1**: Sigma-Aldrich, F6001; **2**: Chem Space; **3**: Aldrich, 452866; **4**: Chem Space; **5**: Chem Space; **6**: Sigma-Aldrich, S567299; **7**: Chem Space; **8**: Chem Space; **9**: synthesized as previously published^14^ **10**: Chem Space; **11**: Chem Space; **13**: Chem Space. For Characterization data of compounds purchased from Chem Space, see Figs. S5-S20. Compound **6** was purchased with no guarantee of identity or purity and was thus characterized after purchase (See NMR and LC in Figs. S21, S22). Physiochemical properties were calculated with the Molinspiration property calculation toolkit (Molinspiration Chemoinformatics, see Table S1).

### Zebrafish husbandry.

All zebrafish experiments were conducted in accordance with approval by the University of Pennsylvania Institutional Animal Care and Use Committee (IACUC) and performed according to ARRIVE guidelines. Adult animals were housed and maintained at the University of Pennsylvania’s aquatic facility, and larvae were reared in a satellite facility, both of which are overseen by the University Laboratory Animal Resources. Adult mating pairs of Tübingen long fin (TLF) zebrafish were routinely cycled to ensure biological diversity of clutches. Fish were kept on a 14-h light/10-h dark cycle at 28°C in E3 embryo water (5 mM NaCl, 0.17 mM KCI, 0.33 mM CaCl_2_, 0.33 mM MgSO_4_, pH 7.4) until 5 days post fertilization (dpf). After experiments, euthanasia by rapid cooling was conducted in accordance with IACUC protocols.

### Zebrafish behavior and compound toxicity.

Behavioral experiments (screening, EC_50_ curves) were performed as previously reported.^[16]^ Antagonism after induction was conducted in a similar manner with different timepoints and solution changes as indicated. The DanioVision Observation Chamber (Noldus) and Ethovision XT16 software were used to track and record (1280 × 960 resolution at 25 frames per second) larval zebrafish (5 dpf) movement in glass 96-well plates (Zissner North America 3600500). All experiments were performed at 28°C with the DanioVision Temperature Control Unit. Zebrafish were allowed to equilibrate in E3 and 2% DMSO (with or without drug) for 10 minutes before being placed in the observation chamber for 22 minutes of recording. Two different endpoints were used to assess depth of anesthesia: spontaneous movement (SM) and elicited movement (EM). In these measures, fish movement was considered to be non-anesthetized. During each observation, fish were given time to acclimate to the chamber, and minutes 15–19 of the recording were used for analysis of SM. An EM response using the Daniovision tapping device (8 out of 8 intensity, movement response measured for 1 second after stimulus) was also measured at the end of the SM observation period. All recordings were scrutinized by a blind observer for technical errors in tracking to ensure accuracy of zebrafish detection and inaccurate tracking was excluded from further analysis. Toxicity was measured in separate experiments by exposing larvae to compounds for 30 minutes, transferring to fresh E3, and then observing mortality after exposure. LD_50_ values were calculated from 24-hour survival.

### Drug preparation and administration.

All concentrated stocks of propofol (Sigma-Aldrich D126608) and library compounds were made in DMSO (Fisher BP231-1) and stored at −20°C. Final concentration of solutions for administration to fish were in E3 with 2% DMSO. Special care was taken in preparation of the sevoflurane (AbbVie Inc.) and isoflurane (Piramal Critical Care) solutions given their high vapor pressures (see Supplemental Methods and Figs. S23, 24). Due to the hydrophobic nature of all library compounds and anesthetics tested, all solutions were stored in glass vials and glass 96-well plates were used for all behavioral experiments.

### Statistical analysis.

All experiments were replicated with either three technical replicates or at least three biologic replicates (separate clutches on separate days with 12 fish per condition per replicate). For most experiments, 5 biologic replicates were conducted generating 50–60 data points from individual fish per condition. All statistical analysis was performed with GraphPad Prism version 10.1.1. ROUT test for outliers were performed with the default Q value of 1%, RAW-One-way ANOVA with Sidak's multiple comparison test. All EC_50_ and IC_50_ curves were fitted in Prism with the model Absolute EC_50_, X is log(concentration) with baseline constraint equal to zero.

### 1-Aminoanthracene (1-AMA) competition fluorescence assay.

The assay was performed as previously described.^[15]^ See Appendix Methods for further detail.

## Data Availability

The datasets generated during and/or analyzed during the current study are available from the corresponding author on reasonable request.
